# Basic Methods of Medical Research (2^nd^ edition: 2008)

**Published:** 2008-07

**Authors:** Abhaya Indrayan

‘*Basic Methods of Medical Research*’ focuses, as the title suggests, on the building blocks of medical research. Conduction of ‘good’ medical research is dependent on the detailed understanding and appropriate handling of ethical, epidemiological, biostatistical, and common sense skills. While it is acknowledged that medical research, like all branches of medicine, is best learnt through ‘doing’ rather than ‘reading’; the availability of a simple text that highlights the conceptual issues is a welcome need. The expectation from such a text would be to convey the fundamental research ethos into the daily practice of even novice researchers. The book should be easily readable and must judiciously emphasize on the mathematical concepts. It should strive to balance between the concepts and the excessive and sometimes unnecessary use of detailed statistics.

The book title suggests the author's intention to acquaint the readers with the basics and sets the tone for an interesting reading, However, the title understates the contents of the book, which truly speaking, are much wider than just ‘basic’. (The author, however, does clarify that the word ‘basic’ is a personal and subjective opinion of the term.) The book is intended for postgraduate and doctoral students who plan to handle medical data. The book does appear to satisfy this intention and does not seem to need a prior experience with data handling. The book is characterized by a clear highlight of the key concepts at the start of each chapter and an exhaustive up-to-date reference list at the end. Liberal re-capitulation of the salient features has been included as boxes within the write-up.

The chapters flow in a sequence and there is a progressive build-up in the contents. Thresholds of normal: disease, clinical, and statistical are well elaborated. The handling of the concept of predictive values and the subsequent discussion on Bayes' rule is commendable. The author explains these difficult concepts with great simplicity. Effect measures are also discussed in great detail and include a small section on number needed to treat. Robustness and sensitivity analysis are a welcome addition. The author has successfully devoted attention to epidemiologic concepts and their role in research methods through this book. Most concepts have been dealt with judiciously barring a few.

Validity: face, construct, criterion, and content could be elucidated with simpler examples. The section on non-parametric methods could be devoted more attention. The index is the weak point in the book due to the over-inclusion of terms. Many terms, totally unrelated with the title and genre, find inclusion in the index.

Overall, priced at a modest Rs. 299, it combines detail with cool simplicity. A must have for all those interested in pursuing medical research.

